# *In-Vivo* Measurement of Ocular Deformation in Response to Ambient Pressure Modulation

**DOI:** 10.3389/fbioe.2021.759588

**Published:** 2021-11-15

**Authors:** Sabine Kling

**Affiliations:** OPTIC Team, Computer Vision Laboratory, ITET Department, ETH Zürich, Zürich, Switzerland

**Keywords:** corneal biomechanics, optical coherence tomography, ambient pressure modulation, *in vivo*, ocular imaging

## Abstract

A novel approach is presented for the non-invasive quantification of axial displacement and strain in corneal and anterior crystalline lens tissue in response to a homogenous ambient pressure change. A spectral domain optical coherence tomography (OCT) system was combined with a custom-built set of swimming goggles and a pressure control unit to acquire repetitive cross-sectional scans of the anterior ocular segment before, during and after ambient pressure modulation. The potential of the technique is demonstrated *in vivo* in a healthy human subject. The quantification of the dynamic deformation response, consisting of axial displacement and strain, demonstrated an initial retraction of the eye globe (−0.43 to −1.22 nm) and a subsequent forward motion (1.99 nm) in response to the pressure change, which went along with a compressive strain induced in the anterior crystalline lens (−0.009) and a tensile strain induced in the cornea (0.014). These mechanical responses appear to be the result of a combination of whole eye motion and eye globe expansion. The latter simulates a close-to-physiologic variation of the intraocular pressure and makes the detected mechanical responses potentially relevant for clinical follow-up and pre-surgical screening. The presented measurements are a proof-of-concept that non-contact low-amplitude ambient pressure modulation induces tissue displacement and strain that is detectable *in vivo* with OCT. To take full advantage of the high spatial resolution this imaging technique could offer, further software and hardware optimization will be necessary to overcome the current limitation of involuntary eye motions.

## Introduction

Biomechanical properties of optical tissues provide important information for both, the pre-operative assessment of patients undergoing refractive surgery and the follow-up of progressive diseases such as keratoconus. Up until today, different approaches have been developed to assess corneal biomechanics in a clinical setting, yet each of them has a different drawback. The earliest clinically applied techniques are based on air-puff induced macroscopic corneal deformation. These techniques rely on geometrical measures extracted from the deformation response, including among others time of corneal applanation (Ocular Response Analyzer) and maximal indentation depth (Corvis ST), to estimate corneal stiffness. However, their strong dependency (Kling and Marcos, 2013) on intraocular pressure (IOP) and corneal thickness apart from mechanical tissue properties demand for sophisticated methods for correction before measures are useful for clinical interpretation. Brillouin microscopy (Scarcelli et al., 2013) is a more recent technique that relies on a non-linear optical scattering effect that is related to the longitudinal elastic modulus. The measured shift in optical wavelength however is also dependent on refractive index and density, which is the reason why measurements are biased by the hydration level of the tissue and in consequence are subjected to diurnal variation (Shao et al., 2018) questioning its usefulness as an objective measure. Optical coherence elastography (OCE) is yet another technique that assesses micro-scale corneal displacement in response to minimal corneal deformation induced either by an array of micro air-puffs (Wang and Larin, 2014), or during corneal applanation with a lens (De Stefano et al., 2018). A disadvantage of the latter two approaches is that measurements are lengthy and uncomfortable to patients. In addition, inherent to all previously proposed methods for biomechanical characterization is that they apply non-physiological loading conditions and thus their clinical relevance remains unclear. More recently, low-amplitude pressure modulation has been suggested as a suitable approach to induce a homogenous and nearly physiological ocular loading condition during OCE measurements (Kling et al., 2019; Kling, 2020). Furthermore, by computing the spatial gradient of tissue deformation in OCE images, mechanical strain can be visualized. Tissue strain is a more direct measure of material stiffness than its geometrical deformation response to a localized macroscopic force. In particular, tissue strain under close-to-physiologic loading conditions is considered relevant for the evaluation of mechanical stability in the context of refractive surgery, degenerative diseases and follow-up after treatments such as corneal cross-linking.

So far, corneal elastography with low-amplitude ambient pressure modulation has been applied either invasively by inserting a needle into the eye globe (Kling et al., 2019), or non-invasively by placing the enucleated eye globe into a pressure chamber (Kling, 2020)—and hence was restricted to *ex vivo* samples. However, from a physical point of view, the stress of a pressurized vessel is only dependent on the pressure difference between interior and exterior pressure. Thus, modulating the ambient pressure has the same effect as modulating the intraocular pressure. In fact, in glaucoma research, the *in vivo* application of a negative pressure to the periorbital region has previously been applied in the context of assessing deformations in the optic nerve head (Midgett et al., 2019), and as a treatment modality to reduce IOP in glaucoma patients (Ethier et al., 2020). In this work, a novel method of employing dynamic ambient pressure modulation *in vivo* while conducting OCT elastography in a human subject is proposed and validated.

## Methods

### Instrument Implementation

A commercial anterior segment swept-source optical coherence tomography (OCT) system (Anterion, Heidelberg Engineering, Heidelberg, Germany) was combined with a customized pressure modulation unit to capture cross-sectional scans of the eye, both before, during and after ambient pressure decrease. The OCT system uses a wavelength range of 1,200–1,400 nm and has an axial resolution of <10 μm in tissue. Its driving software was modified in collaboration with the manufacturer in order to collect repeated recordings of up to 256 subsequent B-scans and get access to raw data. For the measurements presented here, OCT scans consisting of 1222 (axial) × 768 (lateral) pixels and 128 subsequent B-scans were recorded. The duration of the overall scanning time can be approximated by considering the A-scan rate of 50 kHz and a dead-time of ∼1/3 required for mirror repositioning and stabilization before starting a new cross-sectional B-scan. This results in a ∼2.56 s recording period for a single measurement.

**FIGURE 1:**
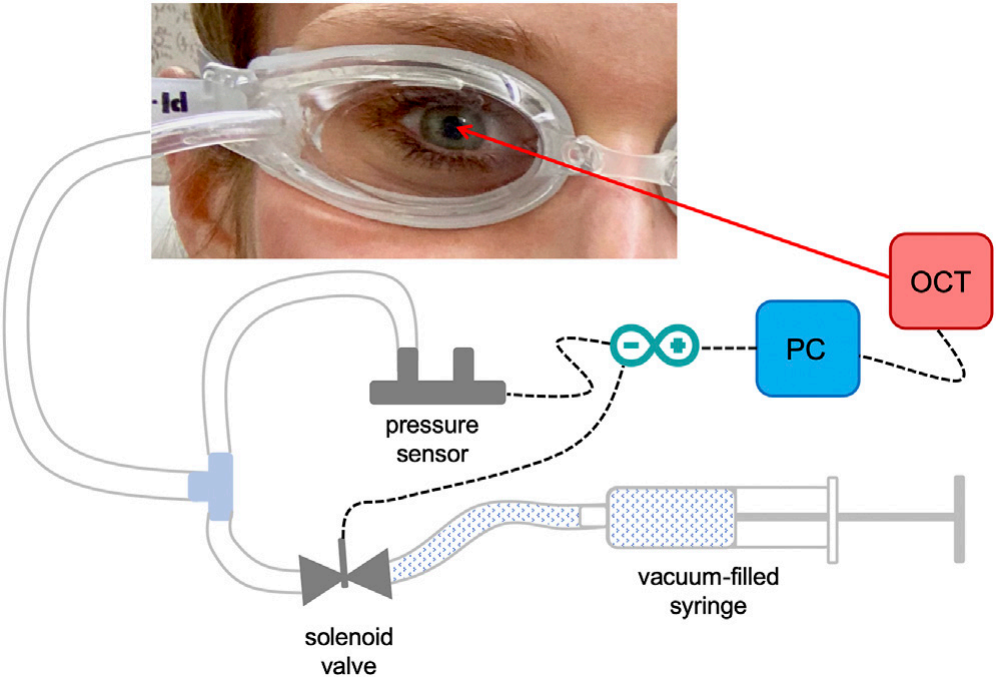
Detail of the set-up used for the measurements. Ambient pressure modulation is performed by triggering the opening of the solenoid valve, which is connected to a vacuum-filled syringe, during the OCT measurement. The differential pressure sensor records the induced pressure modulation, which is in the order of diurnal physiologic IOP variations.

The pressure modulation unit (see Figure 1) consisted of a pair of swimming goggles in which a lateral opening was drilled in order to connect the eye chambers via a flexible tube system to a differential pressure sensor (PA-100-100D-S, Nidec Copal Electronics, Eschborn, Germany) and a solenoid valve that can be triggered to open a connection to an empty 5 ml syringe. In a pre-experiment, sucking a vacuum of 1 ml in the syringe prior to starting the measurement, induced upon opening the valve an immediate ambient pressure decrease in the eye chamber of −5.30 ± 0.36 mmHg (after 100 ms) that slowly increased to −3.78 ± 0.46 mmHg (after 1 s).

The synchronization of the OCT measurement and triggering of the pressure decrease within the time window of OCT recording was implemented using a microcontroller board (Arduino nano, Adafruit Industries).

### Eyes and Measurements

Measurements were conducted in one eye of a healthy, contact lens wearing 34-year old subject *in vivo*. Two different pressure loadings were investigated: a low pressure range corresponding to 1 ml of vacuum (*n* = 3), and a high pressure range corresponding to 3 and 5 ml of vacuum (*n* = 3, each). OCT scans were performed in a similar way as for clinical examination, except that the subject was wearing the customized swimming goggles during the measurements. The experiments were approved by the responsible Institutional Review Board (Ethikkomission ETH Zürich) and followed the tenets of the Declaration of Helsinki. The subject was aware of the nature of the study and signed a consent form.

### Finite Element Modelling

The purpose of the finite element simulation was to reproduce the experimental set-up and facilitate interpretation of the measurement data. An axisymmetric FEM was built in Ansys (Mechanical APDL, V19.2, ANSYS, Inc., Canonsburg, PA, United States) software simulating the experimental set-up. The geometry consisted of the outer ocular shell, the crystalline lens, the ciliary body and its surrounding extraocular parts, representing fatty tissue and ocular muscles. All of these tissues were modelled as structural solids using PLANE183 elements. The aqueous and vitreous humors were modelled with an axisymmetric harmonic acoustic fluid using FLUID29 elements, which were subjected to a pressure of 15 mmHg to represent the intraocular pressure. The complete model consisted of 2145 elements with a maximal size of 400 μm side length. Mesh sizes between 150 and 900 μm were assessed in order to find the best compromise between a stable solution and computational time. The eye globe was dimensioned to have an axial length of 22 mm and a transverse/sagittal diameter of 23 mm, matching roughly the human eye ball (Bekerman et al., 2014). Corneal and scleral tissues were approximated with a linear elastic material model. E-moduli of 1.3 and 6.0 MPa were assigned to cornea and sclera, respectively, along with a density of 1,062 kg/m^3^ in agreement with previous literature (Wollensak et al., 2003; Wollensak and Spoerl, 2004). Poisson’s ratio of the cornea was set to −0.49 representing an auxetic material in order to reproduce the experimentally observed positive strain. The crystalline lens was assigned an E-modulus of 100 kPa and a Poisson’s ratio of +0.49, in agreement with previous literature (Besner et al., 2016). An important part of the model geometry was the extraocular tissue, as it formed the boundary condition of the eye globe when subjected to an under-pressure applied from the side of the anterior ocular surface. Different stiffness values between 5 kPa and 100 MPa were tested to explore the importance of the boundary condition on the observed deformation behaviour. In another scenario, the whole ocular shell was assigned being a rigid object, such that the sole contribution of the extraocular tissue could be quantified. A surface pressure of −3.78 and −8.60 mmHg, respectively, was applied to the anterior surface of the cornea and part of the sclera in order to replicate the loading condition within the swimming goggles during low- and high-pressure modulation. A static analysis type was used for model solving. This is reasonable, as the main purpose of the simulation was to evaluate the role of the extra ocular material properties on the observed deformations and strains in corneal and lens tissues. Moreover, viscoelastic properties have been neglected here and, theoretically, there should not be a steady pressure decline after the sudden ambient pressure reduction (step change).

### Data Analysis

Custom routines were developed in Matlab (The MathWorks, Inc., Natick, MA, United States ) software to process the acquired stack of OCT cross-sectional scans. Using the complex OCT signal, a similar vector-based phase-sensitive tracking approach was adopted as reported recently (Zaitsev et al., 2016; Kling et al., 2019) in order to determine corneal displacement and strain induced in two subsequent scans. The only difference is that previously subsequent B-scans were compared, while in here subsequent A-scans were used due to the inevitable (physiologic) motion *in vivo*. Briefly, the angle of the complex cross-correlation $R=B_{\text{s}}\left(z,x\right)\cdot B_{\text{s}}^{*}\left(z,x+1\right)$ was used to determine axial displacement Δz (in direction of the optical beam) according to:

$$\Delta z=\frac{\lambda \cdot ∠R}{4\pi \cdot n}$$ (1)

where *B* represents an OCT B-scan, B* its complex conjugate, s = {1, 128} is the number of B-scans, λ=1300 nm the mean wavelength of the OCT and *n* = 1.375 the refractive index of ocular tissues. Strain was approximated as the axial gradient and computed by applying a second cross-correlation with the by 1-pixel axially shifted first complex cross-correlation:

$$\varepsilon_{z}=\frac{\lambda \cdot ∠\left(R_{\text{s}}\left(z,x\right)\cdot R_{\text{s}}^{*}\left(z+1,x\right)\right)}{2\pi \cdot n\cdot \text{asu}}$$ (2)

where asu=11.46 μm is the axial sampling unit. Because of involuntary eye motions occurring within the measurement duration, the ocular deformation within the whole pupil size was laterally averaged and the accumulated temporal displacement and strain curves were determined by summing up the corresponding value up for each point in time. Before lateral averaging, corneal surface was detected and used to convert the image into a flat sample.

## Results

### Example Images

**FIGURE 2:**
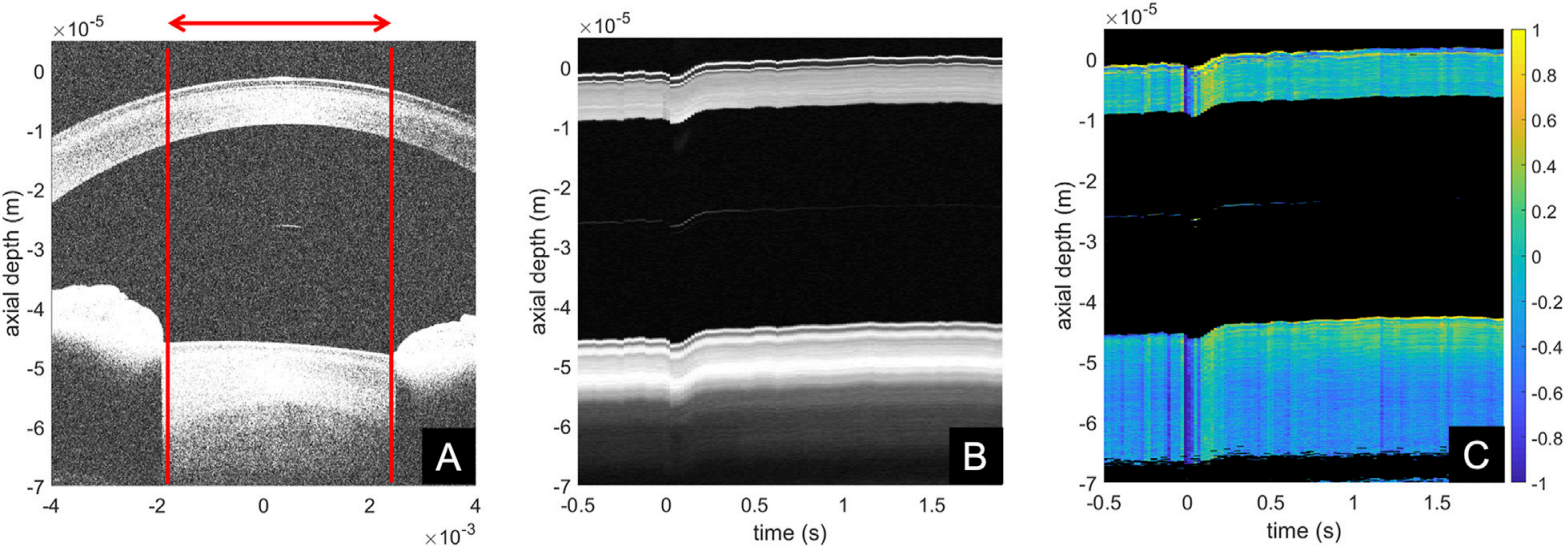
Examples of anterior segment **(A)** structural cross-sectional B-scan, **(B)** structural temporal M-scan representation, **(C)** phase difference temporal M-scan representation.

Figure 2 shows representative images of the different processing steps. Panel A presents a standard structural B-scan of the anterior ocular segment, containing part of the cornea, anterior crystalline lens and iris. The red lines indicate the region that has been averaged for the temporal interpretation. Panel B presents the temporal evolution of the structural signal. At time zero, the solenoid valve was opened. Even though the ambient pressure in the surrounding of the eye was decreased, the eye globe experienced an initial retraction in response to this pressure change, which was followed by a forward movement. Panel C shows the corresponding phase term of the first cross-correlation. It is evident that the initial backward motion of the eye resulted in a negative and its recovery in a positive phase value. Note this phase change is the immediate response at a given time instant, thus small steady changes are difficult to recognize. Despite, the image shows that after 0.2 s the anterior lens moves forward, while the mid lens moves back. The axial gradient of panel C corresponds to compressive and tensile strains.

### Cumulative Analyses

**FIGURE 3:**
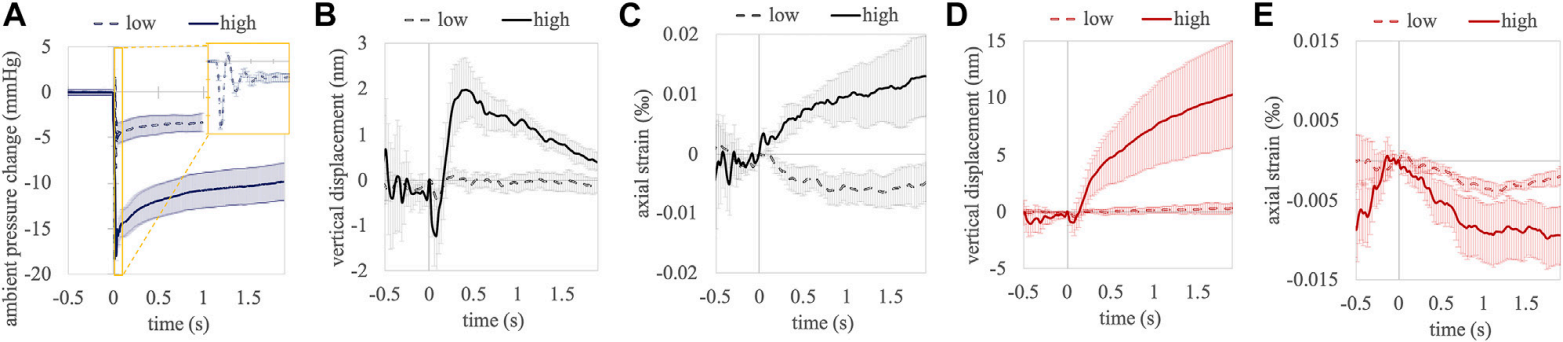
Temporal evolution of ambient pressure **(A)**, accumulated vertical displacement **(B)** and accumulated axial strain **(C)** between subsequent A-scans in the central cornea. Accumulated vertical displacement **(D)** and accumulated axial strain **(E)** in the anterior crystalline lens. Error bars represent standard error.

Figure 3 shows the accumulated temporal response curves of corneal and anterior lens tissue occurring in response to the pressure modulation. Panel A visualizes the pressure change in the eye chamber. At low pressures several periods of pressure oscillations were observed in the 50 ms directly after opening the valve (see yellow inlet), at high pressures this effect was not observed. After the nearly step-wise reduction, the pressure within the eye chamber experienced a slow gradual increase within the measurement period, which corresponds to a decline in the applied pressure difference. Panel B shows the cumulative vertical displacement between two subsequent A-scans as a function of time. Upon pressure change, a backward movement was observed in the cornea, which was more pronounced at a higher (−1.22 ± 1.00 nm) compared to a lower (−0.43 ± 0.22 nm) pressure change. After the initial retraction following a high pressure change, a strong forward motion was observed (1.99 ± 0.71 nm), which was followed by a gradual decline (−0.96 nm/s). This effect was not detectable with a low pressure change. Panel C shows a gradual increase in the accumulated corneal strain after pressure change, which only reached significance with a high pressure change (*p* = 0.002). Panel D shows the cumulative vertical displacement of the anterior crystalline lens. Similar to corneal tissue, an initial retraction was observed with both, a low (−0.47 ± 0.22 nm) and high (−0.95 ± 1.16 nm) pressure change. With a high pressure change however, this backward deformation was largely exceeded by the subsequent steady forward displacement, which increased until the end of the measurement (4.10 nm/s between t = 0.5 and t = 1.9 s). Panel E shows the corresponding strain in the anterior crystalline lens. While negative strains were observed with both, low and high pressure changes, the strain amplitudes were higher in the latter. Table 1 presents displacement and strain values at 0.5 and 1.9 s after the ambient pressure decrease together with a statistical test against zero (one-sample *t*-test, 2-tailed). With a high pressure change, significant strain values were observed in all tissues and at all times except in cornea after 1.9 s, where strain had already recovered its physiologic strain condition. Note that corneal strain with a low pressure change is not significant from zero and therefore, its negative sign is not an indication for different tissue responses at high and low pressures.

**TABLE 1:** Induced deformation 0.5 and 1.9 s after ambient pressure decrease.

			Mean	SD	p-value	Mean	SD	p-value
High ∆P			t = 0.5			t = 1.9		
	Displacement	Cornea	1.446	0.955	**0.008**	0.249	0.476	0.276
	(nm)	Lens	5.526	5.622	**0.006**	10.755	9.628	**0.002**
	Strain	Cornea	0.008	0.004	**0.002**	0.014	0.013	**0.043**
	(‰)	Lens	−0.006	0.005	**0.030**	−0.009	0.007	**0.021**
Low ∆P	Displacement	Cornea	−0.002	0.290	0.990	−0.117	0.293	0.456
	(nm)	Lens	0.045	0.423	0.839	0.285	0.743	0.474
	Strain	cornea	−0.005	0.004	0.063	−0.005	0.006	0.147
	(‰)	Lens	−0.001	−0.001	0.065	−0.002	0.002	0.100

Bold text indicates statistical significance at p < 0.05.

When sitting in front of the OCT system, the induced pressure changes at 1 s showed a higher variation across measurement repetitions than during calibration, when only the swimming goggle was worn: 3.38 (−1.87 to 4.58) mmHg versus −3.78 (−3.22 to 4.34) mmHg, at 1 ml vacuum suction, presented as mean (range). Higher pressure differences had an even wider spread: 8.60 (−5.64 to 9.12) mmHg at 3 ml and −13.4 (−6.99 to −18.5) mmHg at 5 ml. Due to the substantial overlap of pressures achieved with 3 and 5 ml vacuum suction, these two pressures are summarized as “high pressure” in the remaining part of the manuscript, and 1 ml vacuum suction is referred to as “low pressure.”

**FIGURE 4:**
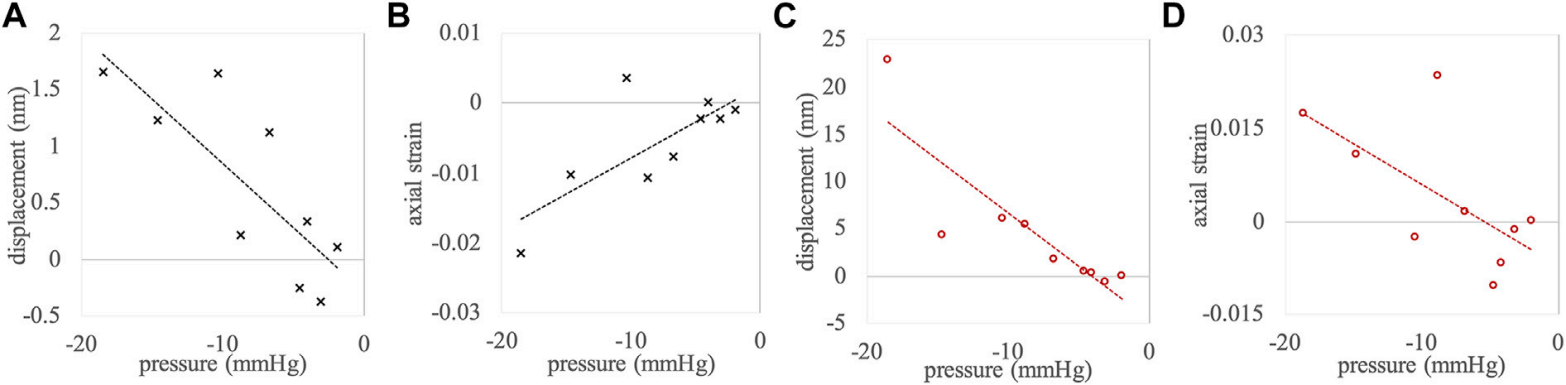
Correlations between pressure change and **(A)** corneal displacement, **(B)** corneal strain, **(C)** anterior crystalline lens displacement, and **(D)** anterior crystalline lens strain. Measurement points represent the complete data set (*n* = 9).

To further analyze the effect of this considerable pressure spread on the extracted parameters, Figure 4 shows correlation plots of axial displacements and strain as a function of ambient pressure change. Table 2 presents the corresponding Pearson correlation coefficients. A clear dependency of the extracted parameters on the ambient pressure change could be confirmed, except in corneal strain, which showed a trend (*p* = 0.057) only.

**TABLE 2:** Pearson correlation coefficients of the pressure change with measured parameters, applied to the complete data set (n = 9).

	Displacement		Strain	
	Cornea	Lens	Cornea	Lens
pressure	−0.81	−0.86	−0.65	0.75
p-value	**0.009**	**0.003**	0.057	**0.020**

Bold text indicates statistical significance at p < 0.05.

### Simulation Results

For better comparison with the FEM simulation, the experimentally measured displacements–representing the induced motion between subsequent A-scans–were converted into an estimated overall deformation $\Delta_{ges}$ , i.e. displacement or strain, by multiplying the maximal measured accumulated deformation $\Delta z_{max}\;$ by the number of A-scans contained in a B-scan $N_{A/B}$ $\left(\Delta_{ges}=\Delta_{max}\cdot N_{A/B}\right)$ . This estimation assumes that the deformation was constant across the time required for a single B-scan, which is reasonable 1) as the experimental data has been averaged across the entire optical zone, and 2) as the deformation occurred over more than one B-scan. The resulting overall displacement with high pressure modulation was 1.5  μm and 8 μm in corneal and lens tissue, respectively. Note that lens deformation may not only result from the external pressure application, but also from accommodation. For the purpose of this study, therefore more focus was put on corneal deformation. The resulting overall strain with high pressure modulation was 10.0 and 7.3‰ in corneal and lens tissue, respectively.

**FIGURE 5:**
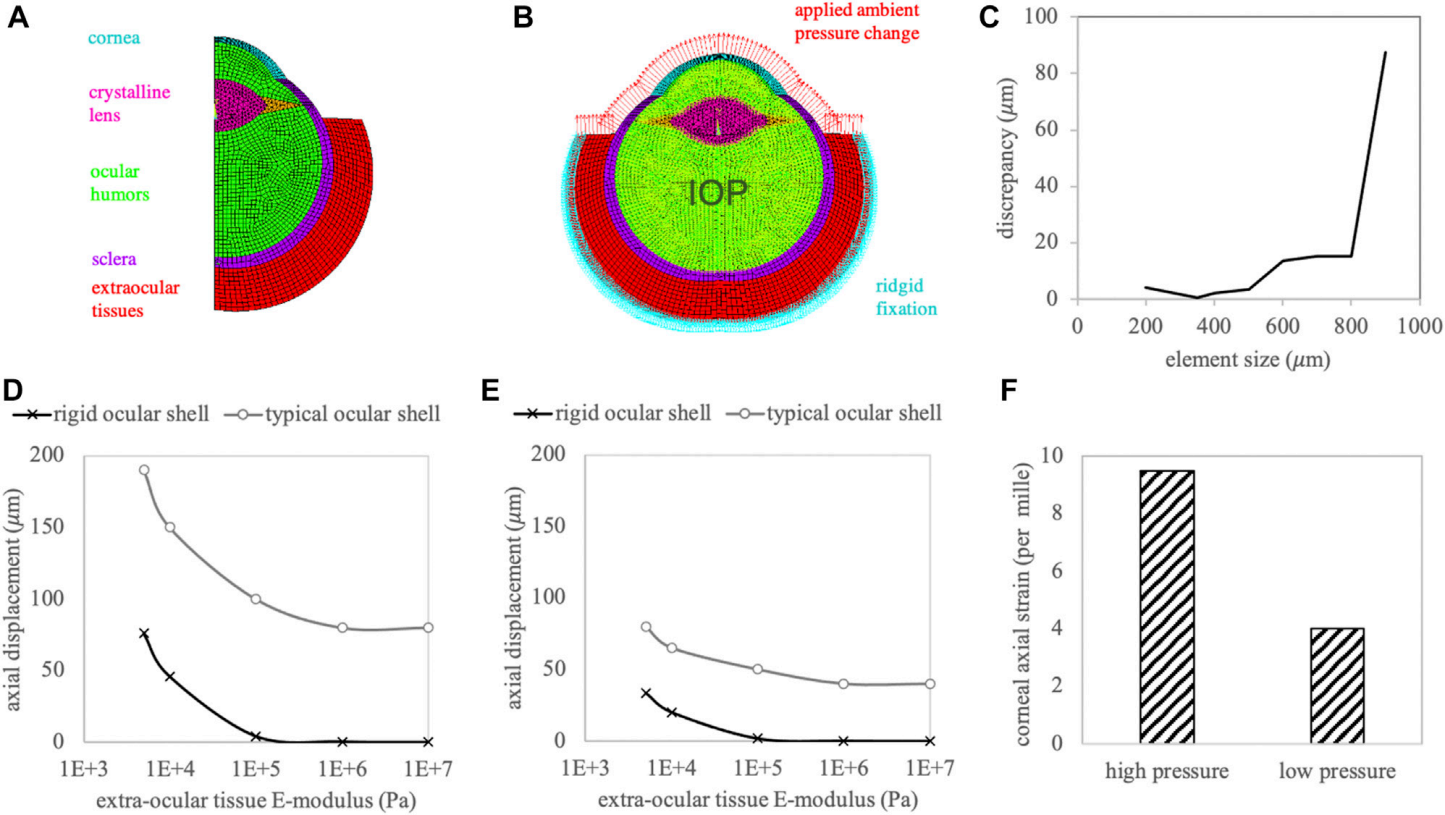
**(A)** Meshed and labelled geometry of the finite element simulation. **(B)** Applied boundary conditions. Red arrows represent the actuation of the ambient pressure modulation. **(C)** Mesh size evaluation. Resulting axial corneal displacement with **(D)** high and **(E)** low ambient pressure modulation. **(F)** Axial corneal strain amplitude.

Figure 5 presents the meshed and labelled model geometry (panel A), the applied boundary conditions consisting of rigid fixation at the outer extraocular tissue, fluid pressure on the contained liquid representing the IOP, surface pressure on the anterior surface representing the test load (panel B), the mesh size evaluation (panel C), as well as the resulting axial displacement (panels D, E) and strain (panel F) in the central corneal tissue. Corneal strain was only dependent on the amount of the applied ambient pressure modulation and not on the stiffness of the extraocular boundary tissue. Similarly, the deformation of the ocular eye globe alone (difference between the two curves in panels D and E) was similar in all cases. Therefore, the extraocular deformation appears to be independent of the eye globe deformation, and the measured deformation the accumulation of both. Over the whole range of the extraocular stiffness values, the axial displacement resulting from the eye globe deformation dominated over the displacement from the extraocular tissue.

## Discussion

This study presents a proof-of-principle that low-to medium-amplitude ambient pressure modulation within a swimming goggle induces ocular displacement and strain *in vivo* that can be detected with OCT. In contrast to previous non-contact measurement approaches developed to assess corneal biomechanics (Luce, 2005; Eliasy et al., 2019), this new technique provides a series of advantages: First, it allows a more homogenous mechanical loading that is similar in amplitude and distribution to the natural stress condition in the eye and thus provides a realistic representation of the physiologic tissue response. For comparison, the localized air-puff applied in non-contact tonometry uses substantially higher pressure loads (Kling et al., 2014) of up to 112 mmHg and induces a large localized deformation stressing the tissue inversely to the IOP, leading to anterior compression and posterior tension (Ariza-Gracia et al., 2015). Second, measurements are fast and can be conducted with a standard OCT device. Third, with some further hardware and software optimization, the technique has the potential to record high-resolution maps of spatial corneal strain distribution. In the current study, data has been laterally averaged across the whole optical zone to reduce measurement noise. In an earlier study, subsequent B-scans were used for phase difference calculation (in contrast to subsequent A-scans in the current study), which permitted to resolve the localized strain field resulting from patterned corneal cross-linking (Kling, 2020). While this analysis has been attempted here as well, it turned out that involuntary eye-motions were too large and moved the reference voxel out of the imaging plane such that the required information was lost. In order to still translate the proposed technique from *ex vivo* experiments into clinical application, a compromise was made computing the phase difference between subsequent A-scans. The advantage of this approach is that the reference scan still shares a relevant part of its voxel with the next scan, if the cross-sectional B-scan is slightly oversampled. In the current study 768 A-scans were contained in a B-scan that span over a range of 8 mm. With an approximate lateral resolution of 30 μm, a pixel-overlap of 65% was reached. Furthermore, phase-wraps will not occur when computing this phase difference, as the deformation possible within 20 μ s (time of 1 A-scan) is too small and therefore, there is no 2π -uncertainty in the detected displacement. A disadvantage of the technique however is that due to the small displacements, the expected signal is close to the level of phase fluctuations inherent to the light source. As a consequence, 1) a higher pressure difference is needed than in *ex vivo* conditions and 2) strain needs to be accumulated over several scans before it becomes detectable. The current study found that strain required at least 21 B-scans to become significant.

Corneal displacement was less noisy than strain and thus easier to detect, however demonstrated a larger standard deviation between measurement repetitions. The fact that the cornea and anterior lens experienced a similar amount of initial retraction in response to the pressure modulation suggests that this behavior reflects a whole eye globe motion. Interestingly, the eye globe was initially retracted even though the pressure in the chamber was decreased. While the exact mechanism behind is not fully understood, it might be related to the swimming goggles getting deeper sucked into the eye socket. After the initial retraction has recovered, a forward displacement was observed in the crystalline lens that steadily increased during the remaining measurement time. In contrast in the cornea, after a strong forward displacement the deformation steadily decreased in the remaining part of the measurement window. Previous literature on the application of negative pressure to the periorbital region (Ethier et al., 2020) described a two-phase response, composed of a rapid increase in ocular shell volume, likely due to blood entering the eye, followed by a slower change, mostly depending on aqueous humor dynamics. Even though the timescale in the current study was substantially smaller (∼2 s versus 20–80 min), it is feasible that blood flow might have contributed to the observed deformations. Despite corneal strain in the crystalline lens could be measured with the current measurement set-up, it needs to be considered that accommodation might have occurred within the measurement interval. Experimental control of this effect in future studies is required in order to make crystalline lens biomechanics accessible by elastography. While the overall positive sign of the ocular displacement behavior is expected from the negative pressure application, the fact that cornea and crystalline lens demonstrated opposite displacement dynamics indicates that the ambient pressure change induced actual deformation and not merely whole eye globe displacement. Furthermore, the significant correlation found between the ambient pressure change and the experimentally induced displacement and strain further confirm the validity of the proposed measurement approach.

The results obtained from FEM simulation demonstrate the independence of the induced corneal strain from extraocular boundary conditions and confirm the general trend observed in the experiments: The eye experiences a forward motion upon ambient pressure decrease, along with a positive and negative strain in the cornea and lens, respectively. Nonetheless, the deformation amplitude in the simulation was substantially larger than what was expected from the experimental values (80–190 μm versus 1.5  μm )—particularly with respect to the deformation of the eye globe. In contrast, strain values were more similar between simulation and experiments (9.5 versus 10.0‰). It is hypothesized that the discrepancy observed in displacement can be attributed geometrical effects. Even though care was taken to use representative dimensions of a human eye globe, a slightly less spherical ocular shell could have led to a larger displacement in the simulation. In addition, the assigned material properties in the model may have further contributed to a discrepancy between experiments and simulation.

A limitation of the current set-up was the variability in the amplitude of the induced ambient pressure change, even though the same volume of vacuum suction was applied. The reason of this may likely be attributed to the fitting of the swimming goggles, which possibly varied in how well the eye chamber sealed with the skin. One way to prevent this issue in the future could be to apply a small under-pressure right from the beginning of the measurement in order to verify the goggle has been correctly placed. A further limitation arises from the current OCT imaging settings. This study used a commercially available spectral domain OCT system with standard imaging settings. Due to involuntary saccadic eye motions, the phase difference between two subsequent B-scans could only scarcely be retrieved and thus was unsuitable for interpretation. Another limitation is the induction of dispersion in the OCT images by the goggle glass, which manifests as a loss of axial resolution. While this effect was only minor (compare Figure 2A) and therefore not specifically addressed, it is not expected to affect displacement or strain estimation as these only depend on the difference between two subsequent scans. A final limitation is the fact that the intraocular pressure of the subject was not measured. As a determinant of pre-stress, the intraocular pressure defines the location of the stress-strain curve that is evaluated during the measurement. Given that corneal tissue has been reported to have non-linear material properties, the same ambient pressure change will lead to a smaller strain in an eye with high than with low intraocular pressure. The key difference to other technologies assessing corneal biomechanics *in vivo* however is that Δ strain is directly accessible. In combination with the applied pressure difference ( Δ stress) and the measured corneal thickness, the tangent elastic modulus can be computed without any further assumptions–and without the need of knowing the intraocular pressure. To further optimize the current measurements, one might increase the exposure time to increase the strain between subsequent A-scans and consequently improve the signal to noise ratio, or increase the imaging speed such that strain computation between subsequent B-scans becomes possible.

## Conclusion

The combination of a customized set of swimming goggles and a standard spectral domain OCT system shows great potential as a clinical tool for non-invasive ocular biomechanical characterization in a close-to-physiologic loading condition. The physiologic condition arises from the fact that the stress in a vessel is only dependent on the pressure difference between interior and exterior pressure (Ibrahim et al., 2015). This implies that ambient pressure modulation is equivalent to invasive intraocular pressure (IOP) modulation. Furthermore, diurnal IOP fluctuations (David et al., 1992) of 5 mmHg are commonly observed in healthy persons and therefore this magnitude of pressure change represents a realistic physiologic loading. The use of a solenoid valve to trigger the change in ambient pressure is a suitable way to enable a rapid loading without losing the phase reference in subsequent OCT scans. The cumulative temporal analyses proved to be sufficiently sensitive to detect tissue displacement and strain induced by the ambient pressure change. These measurements are a first step towards a direct assessment of ocular mechanics involved in refractive surgery and anterior segment ocular disease.

## Data Availability

The raw data supporting the conclusion of this article will be made available by the authors, without undue reservation.
